# The Anti-vaccination Movement: A Regression in Modern Medicine

**DOI:** 10.7759/cureus.2919

**Published:** 2018-07-03

**Authors:** Azhar Hussain, Syed Ali, Madiha Ahmed, Sheharyar Hussain

**Affiliations:** 1 Medicine, Xavier University School of Medicine, Oranjestad, ABW; 2 Psychology, Stony Brook University, Stony Brook, USA; 3 Medicine, Touro College of Osteopathic Medicine, New York, USA; 4 Clinical Psychology, Teachers College, Columbia University, New York, USA

**Keywords:** vaccination, mmr vaccine, measles outbreak, virus, anti-vaccine movement

## Abstract

There have been recent trends of parents in Western countries refusing to vaccinate their children due to numerous reasons and perceived fears. While opposition to vaccines is as old as the vaccines themselves, there has been a recent surge in the opposition to vaccines in general, specifically against the MMR (measles, mumps, and rubella) vaccine, most notably since the rise in prominence of the notorious British ex-physician, Andrew Wakefield, and his works. This has caused multiple measles outbreaks in Western countries where the measles virus was previously considered eliminated. This paper evaluates and reviews the origins of the anti-vaccination movement, the reasons behind the recent strengthening of the movement, role of the internet in the spread of anti-vaccination ideas, and the repercussions in terms of public health and safety.

## Introduction and background

Vaccines are one of the most important measures of preventative medicine to protect the population from diseases and infections. They have contributed to decreasing rates of common childhood diseases and, in some cases, have even wiped out some diseases that were common in years past, such as smallpox, rinderpest, and have nearly eradicated malaria and polio [1]. In fact, according to the World Health Organization’s Polio Global Eradication Initiative, the inactivated polio vaccine (IPV) will be used as a backbone for eradicating poliovirus in the next decade. However, there has been a recent rise in anti-vaccination sentiments surrounding beliefs that vaccines cause more harm than benefits to the health of the children who receive them. The premise of the anti-vaccination movement can also be contributed to the demonization of vaccinations by news and entertainment outlets. Voices such as Jenny McCarthy’s have proven to be influential, sweeping fear and distrust into parents’ minds by parading as “autism experts”. Social media and television talk show hosts, such as Oprah Winfrey, played a big role in this miseducation by giving credence to the campaign. This has caused vaccination rates to sustain a surprising drop in some Western countries [2]. The decrease in vaccinations has led to recent outbreaks of diseases that were thought to be “eliminated”, such as measles. Still, other reasons for the anti-vaccination movement can be due to personal reasons, such as religious or secular views. A drop in immunizations poses a threat to the herd immunity the medical world has worked hard to achieve. Global communities are now more connected than ever, which translates to a higher probability of the transmission of pathogens. The only thing that can protect populations against a rapidly spreading disease is the disease's resistance created by herd immunity when the majority are immune after vaccinations. Given the highly contagious nature of diseases like measles, vaccination rates of 96% to 99% are necessary to preserve herd immunity and prevent future outbreaks [3].

## Review

Origins of the anti-vaccination movement

Fear of vaccines and myths against them are not a new phenomenon. Opposition to vaccines goes as far back as the 18th century when, for example, Reverend Edmund Massey in England called the vaccines “diabolical operations” in his 1772 sermon, “The Dangerous and Sinful Practice of Inoculation” [4]. He decried these vaccines as an attempt to oppose God’s punishments upon man for his sins [5]. Similar religious opposition was seen in the “New World” even earlier, such as in the writings of Reverend John Williams in Massachusetts, who also cited similar reasons for his opposition to vaccines claiming that they were the devil’s work [6]. However, opposition against vaccines was not only manifested in theological arguments; many also objected to them for political and legal reasons. After the passage of laws in Britain in the mid-19th century making it mandatory for parents to vaccinate their children, anti-vaccine activists formed the Anti-Vaccination League in London. The league emphasized that its mission was to protect the liberties of the people which were being “invaded” by Parliament and its compulsory vaccination laws [7]. Eventually, the pressure exerted by the league and its supporters compelled the British Parliament to pass an act in 1898, which removed penalties for not abiding by vaccination laws and allowed parents who did not believe vaccination was beneficial or safe to not have their children vaccinated [8]. Since the rise and spread of the use of vaccines, opposition to vaccines has never completely gone away, vocalized intermittently in different parts of the world due to arguments based in theology, skepticism, and legal obstacles [9].

Anti-vaccination propaganda

While pushback against the measles vaccine due to fears of its connection to autism is the most recent example that comes to mind, there have been other instances of outbreaks of previously “extinct” diseases in modern times. One example is the refusal of some British parents to vaccinate their children in the 1970s and 1980s against pertussis in response to the publication of a report in 1974 that credited 36 negative neurological reactions to the whole-cell pertussis vaccine [10]. This caused a decrease in the pertussis vaccine uptake in the United Kingdom (UK) from 81% in 1974 to 31% in 1980, eventually resulting in a pertussis outbreak in the UK, putting severe strain and pressure on the National Health System [11-12]. Vaccine uptake levels were elevated to normal levels after the publication of a national reassessment of vaccine efficacy that reaffirmed the vaccine’s benefits, as well as financial incentives for general practitioners who achieved the target of vaccine coverage [13]. Disease incidence declined dramatically as a result.

The anti-vaccination movement was most strongly rejuvenated in recent years by the publication of a paper in The Lancet by a former British doctor and researcher, Andrew Wakefield, which suggested credence to the debunked-claim of a connection between the measles, mumps, and rubella (MMR) vaccine and development of autism in young children [14]. Several studies published later disproved a causal association between the MMR vaccine and autism [15-18]. Wakefield drew severe criticism for his flawed and unethical research methods, which he used to draw his data and conclusions [19]. A journalistic investigation also revealed that there was a conflict of interest with regard to Wakefield’s publication because he had received funding from litigants against vaccine manufacturers, which he obviously did not disclose to either his co-workers nor medical authorities [20]. For all of the aforementioned reasons, The Lancet retracted the study, and its editor declared it “utterly false” [21]. As a result, three months later, he was also struck off the UK Medical Registry, barring him from practicing medicine in the UK. The verdict declared that he had "abused his position of trust" and "brought the medical profession into disrepute" in the studies he carried out [22].

Repercussions of declining vaccination rates

The damage, however, was already done and the myth was spread to many different parts of the world, especially Western Europe and North America. In the UK, for example, the MMR vaccination rate dropped from 92% in 1996 to 84% in 2002. In 2003, the rate was as low as 61% in some parts of London, far below the rate needed to avoid an epidemic of measles [23]. In Ireland, in 1999-2000, the national immunization level had fallen below 80%, and in part of North Dublin, the level was around 60% [24]. In the US, the controversy following the publication of the study led to a decline of about 2% in terms of parents obtaining the MMR vaccine for their children in 1999 and 2000. Even after later studies explicitly and thoroughly debunked the alleged MMR-autism link, the drop in vaccination rates persisted [25].

As a result, multiple breakouts of measles have occurred throughout different parts of the Western world, infecting dozens of patients and even causing deaths. In the UK in 1998, 56 people contracted measles; in 2006, this number increased to 449 in the first five months of the year, with the first death since 1992 [26]. In 2008, measles was declared endemic in the UK for the first time in 14 years [27]. In Ireland, an outbreak occurred in 2000 and 1,500 cases and three deaths were reported. The outbreak was reported to have occurred as a direct result of a drop in vaccination rates following the MMR controversy [28]. In France, more than 22,000 cases of measles were reported from 2008 - 2011 [29]. The United States has not been an exception, with outbreaks occurring most recently in 2008, 2011, and 2013 [30-32].

Perhaps the most infamous example of a measles outbreak in the United States occurred in 2014-2015. The outbreak was believed to originate from the Disneyland Resort in Anaheim, California and resulted in an estimated 125 people contracting the disease [33]. It was estimated that MMR vaccination rates among the exposed population in which secondary cases have occurred might be as low as 50% and likely no higher than 86% [34]. Physicians in the region were criticized for deviating from the CDC's (Center for Disease Control and Prevention) recommended vaccination schedule and/or discouraging vaccination. As a result, California passed Senate Bill 277, a mandatory vaccination law in June 2015, banning personal and religious exemptions to abstain from vaccinations [35].

Technology and its effects on anti-vaccination movement

Access to medical information online has dramatically changed the dynamics of the healthcare industry and patient-physician interactions. Medical knowledge that was previously bound to textbooks and journals, or held primarily by medical professionals, is now accessible to the layman, which has shifted the power from doctors as exclusive managers of a patient's care to the patients themselves [36]. This has led to the recent establishment of shared decision-making between patients and healthcare physicians [37]. While this is beneficial in some ways, the dissemination of false and misleading information found on the internet can also lead to negative consequences, such as parents not giving consent to having their children vaccinated. When it comes to vaccines, the false information is plentiful and easy to find. An analysis of YouTube videos about immunization found that 32% opposed vaccination and that these had higher ratings and more views than pro-vaccine videos [38]. An analysis of MySpace blogs about HPV immunization found that 43% portrayed the immunization in a negative light; these blogs referenced vaccine-critical organizations and cited inaccurate data [39]. A similar study of Canadian internet users tracked the sharing of influenza vaccine information on social media networks, such as Facebook, Twitter, YouTube, and Digg. Of the top search results during the study period, 60% promoted anti-vaccination sentiments [40]. A study that examined the content of the first 100 anti-vaccination sites found after searching for “vaccination” and “immunization” on Google concluded that 43% of websites were anti-vaccination (including all of the first 10) [41].

Online anti-vaccination authors use numerous tactics to further their agendas. These tactics include, but are not limited to, skewing science, shifting hypotheses, censoring opposition, attacking critics, claiming to be “pro-safe vaccines”, and not “anti-vaccine”, claiming that vaccines are toxic or unnatural, and more [42]. Not only are these tactics deceitful and dishonest, they are also effective on many parents. A study that evaluated how effectively users assessed the accuracy of medical information about vaccines online concluded that 59% of student participants thought retrieved sites were entirely accurate; however, out of the 40 sites they were given, only 18 were actually accurate, while 22 were inaccurate. These sites were not evidence-based and argued vaccines were inherently dangerous without any merit-based argument. More than half of participants (53%) left the exercise with significant misconceptions about vaccines [43]. Research has also shown that viewing an anti-vaccine website for merely 5 - 10 minutes increased perceptions of vaccination risks and decreased perceptions of the risks of vaccine omission, compared to visiting a control site [44]. The study also found that the anti-vaccine sentiments obtained from viewing the websites still persisted five months later, causing the children of these parents to obtain fewer vaccinations than recommended [45]. The role of the online access to false anti-vaccination information just cannot be understated in examining the rise and spread of the anti-vaccination movement.

Ethical and legal issues regarding vaccination

Opposition to the MMR vaccine among parents leads to an ethical dilemma that can be analyzed using both medical ethics and moral principles. Medical ethics call for health professionals to abide by a code of bioethics upholding autonomy, non-maleficence, beneficence, and justice. The most relevant in mandating vaccinations are autonomy and non-maleficence [46]. Patients are entitled to the right to refuse vaccination using “our children, our choice” based on their autonomy, while health care providers are morally obligated to treat everyone with non-maleficence and avoiding harm to society at all costs.

At the individual level, religion is a common reason to refuse vaccination. The MMR vaccine specifically has been the cause of instigating debate among the Hindu, Protestant, Orthodox Jewish, and Jehovah’s Witness communities. Specific religious views on vaccines in general, however, are not normally the cause for debate but instead the components of the MMR vaccine [47]. The MMR vaccine, combined with the rubella vaccine, was originally derived from the cells of aborted fetal tissue. Hindu, Protestant, Muslim, and Jewish communities are generally opposed to abortion for moral reasons based on religious teachings; thus, individuals from these beliefs may cite religious reasons for filing vaccine exemptions. Further, the MMR vaccine contains porcine gelatin as a stabilizer, a means for ensuring effective storage. The porcine ingredients are unlike gelatins used for oral consumption and purified down to small peptides, commonly used in medicine capsules as well [48]. As there is a wide range of practice preferences in every religion, some individuals belonging to religions, such as Judaism, Islam, and Hinduism (to name a few), may be opposed to injecting a porcine product into their body along with the vaccine [47]. Further, other religious views, such as the ones held by Dutch-Protestant Christian congregations, consider vaccinations “inappropriate meddling in the work of God”. These groups, therefore, believe that we should not change the predestined fate of someone who becomes ill [49].

While exercising autonomy and refusing vaccination is valid for sensitive personal issues, it will cause more harm than good if a certain percentage of the population does not get vaccines causing the immunization rate to fall below the herd immunity threshold. This threshold varies in every disease. The development of vaccines is considered one of the greatest strides made in medicine due to the enormous benefits to an entire population. From an ethics perspective, achieving herd immunity and minimizing the amount of “freeloaders” is in the best interest of society as a whole [48-49].

Further, studies liken the decision to object to vaccinations to military service drafts. For the conscientious objectors, military duty and receiving a vaccine hold the same costs: liberty, personal risk, and utility in terms of time [41]. Naturally, the costs of military duty are more taxing and demand more from an individual than receiving a vaccine. In terms of herd immunity and depending on the severity of impending diseases, these costs are ones that they should incur for the benefit of themselves as well as society.

At the forefront of the legal complications lies the state-regulated vaccinations for all children attending school. Anti-vaccination proponents argue that this is an infringement upon autonomy; however, public health policymakers justify their actions using rule utilitarianism. Rule utilitarianism is the ideology that a rule for society should be established that has the best outcome for the greatest amount of people in the society. In addition to this, John Stuart Mill’s essay, “On Liberty”, explains the Harm Principle that is often used to justify mandated infectious disease control methods, including vaccines [50]. The Harm Principle justifies interfering with autonomy and individual liberties, against their will, if it is done so as to prevent harm to others. An example of this was seen in California in 2014-2015 after an outbreak of measles led to the passing of Senate Bill 277 calling for state-mandated vaccinations for everyone - no personal exemptions. The root of the problem, however, was most likely to be contributed to Wakefield’s fraudulent findings striking the fear of a vaccination-autism link in parents, which led to an all-time low rate of people receiving the MMR vaccine. The hoax has been called the most damaging medical hoax in 100 years after bringing about outbreaks of diseases otherwise eradicated [8-9, 11].

In the times that we have achieved herd immunity, there remain two questions then. Can legal exemptions still be justified? And should these exemptions be limited to religious reasons or should they include secular reasoning as well [21, 25]? Most scientists and medical experts suggest that exemptions should only even be considered if society is well within the limits for herd immunity. As for the religious versus secular debate, it is difficult to ignore secular objections as most of them are rooted in spiritual or holistic personal views [6, 47]. Since herd immunity is cumulative, the ability to waive immunizations is concluded to be difficult but not impossible. If the waivers are given to a small number of individuals who sincerely need them rather than ones who are inconvenienced by them, waivers may be ethically and legally sound. 

## Conclusions

The rise of anti-vaccination movements in parts of the Western world poses a dire threat to people’s health and the collective herd immunity. People of all ages have fallen victim to recent outbreaks of measles, one of the most notable “eliminated” diseases that made a comeback as a direct consequence of not reaching the immunization threshold for MMR vaccines. These outbreaks not only put a strain on national healthcare systems but also cause fatal casualties. Therefore, it is of the utmost importance that all stakeholders in the medical world - physicians, researchers, educators, and governments - unite to curb the influence of the anti-vaccination movement targeting parents. Research has shown that even parents favorable to vaccination can be confused by the ongoing debate, leading them to question their choices. Many parents lack basic knowledge of how vaccines work, as well as access to accurate information explaining the importance of the process. Furthermore, those with the greatest need for knowledge about vaccination seem most vulnerable to this information. Further, we must effectively combat the wrongful demonization of vaccinations through social media and news media platforms. A qualitative study that explored how parents respond to competing media messages about vaccine safety concluded that personal experiences, value systems, and level of trust in health professionals are essential to parental decision making about immunization. Therefore, to combat the anti-vaccination movement, there must be a strong emphasis on helping parents develop trust in health professionals and relevant authorities, educating them on the facts and figures, debunking the myths peddled by the anti-vaccination movements, and even introducing legislation that promotes vaccination, if not mandating it.
